# The influences of gender and aging on optic nerve head microcirculation in healthy adults

**DOI:** 10.1038/s41598-019-52145-1

**Published:** 2019-10-30

**Authors:** Tatsuhiko Kobayashi, Tomoaki Shiba, Ayako Kinoshita, Tadashi Matsumoto, Yuichi Hori

**Affiliations:** 1 Department of Ophthalmology, School of Medicine, Toho University, Tokyo, Japan; 2 Department of Ophthalmology, Japan Community Health Care Organization, Tokyo Kamata Medical Center, Tokyo, Japan

**Keywords:** Predictive markers, Preclinical research

## Abstract

Potential differences in the nature of the influences of aging and gender on the optic nerve head (ONH) microcirculation, using laser speckle flowgraphy (LSFG) were evaluated. We studied 908 healthy subjects (men = 701, age: 50.0 ± 9.1 yrs, women = 208, 49.8 ± 9.5 yrs, p = 0.76). The average, maximum (Max), and minimum (Min) mean blur rate (MBR) in a heartbeat were evaluated. The parameters were analyzed separately for the tissue, vessels, and throughout the ONH (All). We investigated which MBR sections are correlated with gender and age by univariate and multivariate regression analyses. The Max MBR-All (r = −0.31) was most strongly correlated with gender (men = 1, women = 0). The Min MBR-All (r = −0.24) was most strongly correlated with age, followed by Min MBR-All (r = −0.20). The factors contributing independently to the Max MBR-All were gender (β = −0.15), pulse pressure, spherical refraction, ocular perfusion pressure, and red blood cell (RBC) count. The factors contributing independently to the Min MBR-Vessel were gender (β = −0.09), age (β = −0.25), body mass index, heart rate, and spherical refraction. The factors contributing independently to the Min-MBR-All were age (β = −0.22), heart rate, and RBC count. Our results revealed that gender differences influence the Max MBR, and aging influences the Min MBR. These correlations were stronger than that of average MBR.

## Introduction

Laser speckle flowgraphy (LSFG), a noninvasive quantitative method for determining the ocular blood flow^1,2^, is based on the changes in the speckle pattern of laser light reflected from the fundus of the eye^3^. LSFG is dependent on the movement of erythrocytes in the retina, the choroid, and the optic nerve head (ONH), and the mean blur rate (MBR) is an indicator of ocular blood flow^4,5^. It was reported that measurements of MBR shows excellent repeatability with intraclass correlation coefficients and were barely affected by pupil dilation^6^.

The MBR is usually presented as the average value calculated from 118 MBR images (118 frames, 4-sec period) tuned to the cardiac cycle. Several studies revealed that gender differences and/or aging affect the average MBR in the ONH area^6–9^, indicating that age^7–9^ and gender differences^6–9^ are significantly correlated with the average MBR of the entire ONH area. However, the average MBR value is the mean value of the variation of the MBR during the systolic-to-diastolic cardiac cycle, and thus a decrease in the average MBR is caused by a decrease in the maximum MBR or the minimum MBR or both during the cardiac cycle.

We hypothesized that the variations in the average MBR values that are caused by increasing versus decreasing the max. or min. MBR occur in clearly different manners. In addition, the influences of gender differences, aging, and other factors on the max., min., and average MBR in the ONH have not been established. We conducted the present study to clarify the influences of gender differences and aging on the max., min., and average values of the MBR parts in the ONH by examining a large number of healthy subjects.

## Subjects and Methods

### Study design

The Ethics Committee of the Toho University School of Medicine approved this study (no. A16062), which was cross-sectional in nature, and all patients provided informed consent for their participation in accord with the tenets of the Declaration of Helsinki. This study was registered in the UMIN (ID: UMIN000026778).

### Subjects

We studied a total of 1,079 subjects who had participated in a medical checkup program at the Department of Health Care Center of the Japan Community Health Care Organization, Tokyo Kamata Medical Center between December 2016 and December 2018. All of the subjects were Japanese. Patients were excluded if they had atherosclerotic diseases such as hypertension, dyslipidemia, diabetes mellitus, cardiovascular or cerebrovascular events, arrhythmia, or ophthalmic disease (such as glaucoma, uveitis, optic neuropathy, vitreous or retinal disease) or retinal or choroidal vascular disease; best corrected visual acuity <40/50; or if they had undergone a previous intraocular surgery. Eighteen women and 153 men were excluded, and a final total of 908 subjects (701 men, 208 women) met the study criteria.

Blood pressure measurements and LSFG were performed after the patients had rested for 10 min in a quiet, air-conditioned room with the temperature maintained at 24 °C. All of the subjects abstained from smoking, alcohol, and caffeine for ≥24 hr prior to the measurements. All of the evaluations were performed between 9:00 and 11:00 a.m. after the subject had fasted overnight.

### LSFG measurements

LSFG images were obtained by an LSFG-NAVI™ system (Softcare Co., Fukuoka, Japan), and the max., min., and average MBR values were calculated by LSFG Analyzer software (ver. 3.0.47, Softcare). The details of the determination of the LSFG measurements from fundus images were as described^4,5^.

**Figure 1:**
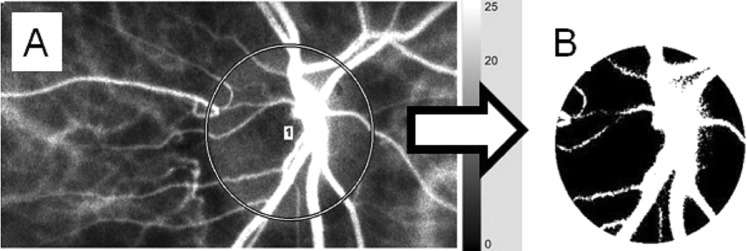
Analyzing the MBR values in the ONH circulation using LSFG. The gray-scale map of the total measurement area. (**A**) The circle surrounds the area of the ONH. (**B**) The software separates out the retinal vessels by using an automated definitive threshold throughout the ONH, within the ONH vessel (shown in *white*), and within the ONH tissue (*black*).

**Figure 2:**
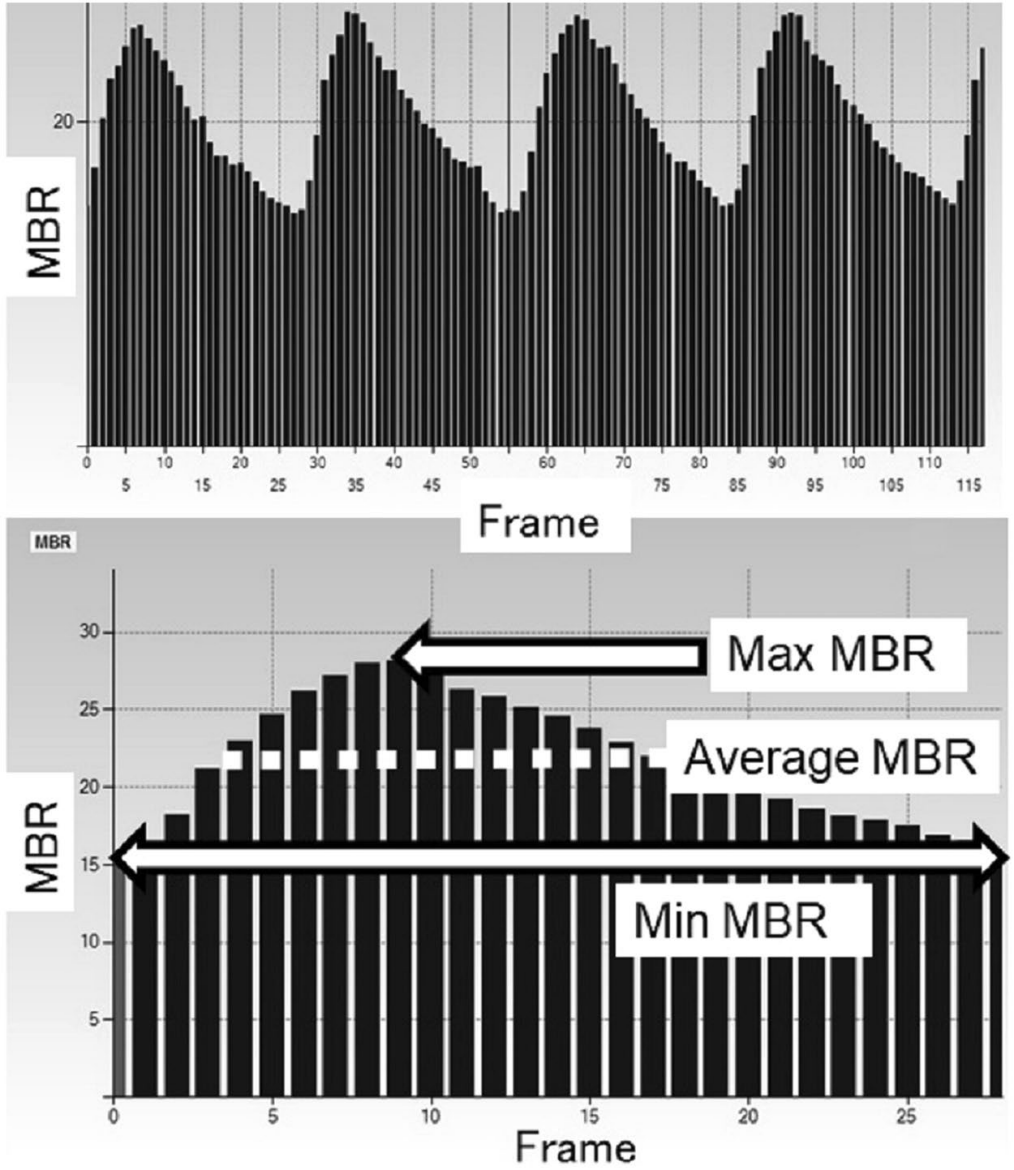
Variation of the MBR, which is tuned to the cardiac cycle for 4 sec. (**A**) The total number of frames was 118. (**B**) The software calculates the normalization of one pulse. Max, Min, and average MBR in the ONH-Vessel, Tissue, and entire ONH (All) are calculated on this screen. Max MBR: The maximum MBR value in a heartbeat. Min MBR: The minimum value of the variation of the MBR in a heartbeat (max. value of steady blood flow). Average MBR: The mean quantity of MBR in a heartbeat.

Briefly, the LSFG consists of a fundus camera equipped with a diode laser (wavelength 830 nm) and a CCD camera. The MBR is derived from the pattern of speckle contrast produced by the interference of the laser scattered by blood cells moving in the ocular fundus^9^. For the evaluation of the ONH area circulation, a circle was set surrounding the ONH (Fig. 1A). The software separated out the vessels using the automated definitive threshold (Fig. 1B). Within a 4-sec period tuned to the cardiac cycle, 118 MBR images (118 frames) were recorded from the circled area. The analysis of the screen (which was normalized to one pulse automatically) is then displayed (Fig. 2), and the analysis of the min., max., and average MBR was conducted on this screen. The max. MBR was determined by the peak of the MBR during one normalized cardiac cycle (Fig. 2). The min. MBR was determined based on the baseline level of the MBR during one normalized cardiac cycle instead of the steady blood flow (Fig. 2). The average MBR was determined based on the mean quantity of MBR during normalized one cardiac cycle. The MBR was analyzed respectively in the ONH tissue (Tissue), in the vessels of the ONH (Vessel), and throughout the entire ONH (All).

All of the measurements were taken with the patient in the seated position, without the use of pupil-dilating eye drops. Only data from the right eye were used for the analyses.

### Measurements of systemic and laboratory parameters

The following values were measured as systemic parameters: age (years), height (cm), weight (kg), waist circumference (cm), body mass index (BMI, kg/m^2^), heart rate (beats per minute, bpm), smoking status, pulse pressure (mmHg), and the mean arterial blood pressure (MABP, mmHg) calculated as the diastolic blood pressure + (systolic blood pressure − diastolic blood pressure)/3.

The laboratory profile of each subject was comprised of the determination of the following from fasting morning blood samples: the white blood cell (WBC) count (×10^3^/μl), red blood cell (RBC) count (×10^4^/μl), platelet count (×10^3^/μl), and the estimated glomerular filtration rate (eGFR, ml/min/1.73 m^2^), which can be obtained using a simple formula from the Japanese Modification of Diet in Renal Disease Study^10^.

### Measurements of other ophthalmic parameters

The spherical refraction (diopters, D), intraocular pressure (IOP, mmHg) measured by non-contact tonometry, and ocular perfusion pressure (OPP, mmHg) calculated from (2/3 MABP) – IOP were assessed as ocular parameters.

### Statistical analyses

Data for the continuous variables are presented as the mean ± SD. The unpaired t-test, Mann-Whitney U-test, and 2 × 2 chi-square test were used for the comparison of subjects’ background parameters. We performed a univariate regression analysis to determine which of the MBR parameters in the ONH are most closely correlated with gender differences and age. We then conducted univariate and multivariate regression analyses to determine the independent factors for the MBR values in the ONH that are most closely correlated with gender differences and age. P-values < 0.05 were accepted as significant. The Stat View program ver. 5.0 (SAS, Cary, NC) was used for the statistical analyses.

## Results

Table 1 summarizes the subjects’ background parameters by gender. The ages of the men (50.0 ± 9.1 yrs) did not differ significantly from those of the women (49.8 ± 9.5 yrs, p = 0.76). The height, weight, BMI, WBC count, RBC count, and smoking rate of the men were all significantly higher than those of the women (p < 0.0001, respectively). The eGFR values in the women were significantly higher than those of the men (p = 0.001). Among the blood pressure measurements, the MABP values of the men were significantly higher than those of the women (p < 0.0001), whereas the pulse pressure in the women was significantly higher than that in the men (p = 0.002). The heart rate did not differ significantly between the men and women (p = 0.32). Of the ophthalmic parameters, the spherical refraction in the men was significantly lower than that in the women (p = 0.002), and the OPP in the men was significantly higher than that in the women (p < 0.0001).

**Table 1:** Clinical parameters of the 908 Japanese men and women.

Parameter Men (n = 701)	Women	(n = 207)	p-value
Age, yrs	50.0 ± 9.1	49.8 ± 9.5	0.76^a^
Height, cm	170.6 ± 6.1	158.5 ± 5.1	<0.0001^a^
Weight, kg	70.0 ± 10.8	56.5 ± 9.2	<0.0001^a^
Waist circumference, cm	84.9 ± 8.9	78.6 ± 8.9	<0.0001^a^
BMI, kg/m^2^	24.1 ± 3.3	22.5 ± 3.5	<0.0001^a^
Heart rate, bpm	70.8 ± 10.5	70.0 ± 9.3	0.32^a^
MABP, mmHg	94.4 ± 13.9	86.4 ± 12.5	<0.0001^a^
Pulse pressure, mmHg	6.3 ± 10.9	49.1 ± 12.5	0.002^a^
Spherical refraction, D	−2.2 ± 2.6	−1.7 ± 2.5	0.002^b^
IOP, mmHg	12.0 ± 2.7	11.7 ± 2.5	0.13^a^
OPP, mmHg	50.9 ± 9.3	46.0 ± 8.2	<0.0001^a^
WBC, ×10^3^/μl	5.9 ± 1.6	5.4 ± 1.5	<0.0001^a^
RBC, ×10^4^/μl	486.9 ± 43.0	436.2 ± 37.4	<0.0001^a^
Platelets, ×10^3^/μl	25.4 ± 5.5	25.0 ± 4.8	0.32^a^
eGFR, ml/min/1.73 m^2^	78.3 ± 15.0	82.1 ± 13.8	0.001^a^
Current smoking (%)	38 (18.4)	235 (33.5)	<0.0001^c^

The data are mean ± SD or number (%). ^a^Unpaired t-test, ^b^Mann-Whitney U-test, ^c^2 × 2 chi-square test. bpm: beat per minute, D: diopters, eGFR: estimated glomerular filtration rate, IOP: intra-ocular pressure, MABP: mean arterial blood pressure, OPP: ocular perfusion pressure, RBC: red blood cell count, WBC: white blood cell count.

The correlation coefficients from the univariate regression analysis between genders, aging, and MBR variables are shown in Table 2. All sections of the average MBR (Vessel: r = −0.17, Tissue: r = −0.14, All: r = −0.24, p < 0.0001, respectively), Max MBR (Vessel: r = −0.26, Tissue: r = −0.21, All: r = −0.31, p < 0.0001, respectively), Min MBR-Vessel (r = −0.07, p < 0.05), and Min MBR-All (r = −0.15, p < 0.0001) were significantly negatively correlated with gender (men = 1, women = 0). The average MBR-Vessel (r = −0.15, p < 0.0001), MBR-All (r = −0.11, p < 0.01), and all sections of the Min MBR (Vessel: r = −0.24, p < 0.0001, Tissue: r = −0.12, p < 0.001, All: r = −0.20, p < 0.0001) were significantly negatively correlated with age, whereas Max MBR-Tissue was significantly positively correlated with age (r = 0.10, p < 0.001).

**Table 2:** Correlation coefficients from the univariate regression analysis between genders, aging and MBR variables.

Objective variable: MBR variables	Gender (men = 1, women = 0)	Age
	r	r
vg MBR-Vessel	−0.17^⁂^	−0.15^⁂^
Avg MBR-Tissue	−0.14^⁂^	−0.03
Avg MBR-All	−0.24^⁂^	−0.11^⁑^
Max MBR-Vessel	−0.26^⁂^	−0.03
Max MBR-Tissue	−0.21^⁂^	0.10^⁑^
Max MBR-All	−0.31^⁂^	0.01
Min MBR-Vessel	−0.07*	−0.24^⁂^
Min MBR-Tissue	−0.06	−0.12^⁑^
Min MBR-All	−0.15^⁂^	−0.20^⁂^

Avg: average, MBR: mean blur rate, Max: maximum, Min: minimum.

^*^p < 0.05, ^⁑^p < 0.001, ⁂p < 0.0001.

We then conducted univariate and multivariate regression analyses to identify factors independently contributing to Max MBR-All, Min MBR-Vessel, and MBR-All (Tables 3 and 4). The results demonstrated that the factors contributing independently to the Max MBR-All (R = 0.40, p < 0.0001) were gender (men = 1, women = 0: β = −0.15, p < 0.001), pulse pressure (β = 0.13, p < 0.0001), spherical refraction (β = 0.08, p < 0.05), OPP (β = −0.14, p < 0.001), and the RBC count (β = −0.17, p < 0.0001). The factors contributing independently to the Min MBR-Vessel (R = 0.35, p < 0.0001) were gender (β = −0.09, p < 0.001), age (β = −0.25, p < 0.0001), BMI (β = 0.09, p < 0.05), heart rate (β = 0.17, p < 0.0001), and spherical refraction (β = 0.14, p < 0.0001). The factors contributing independently to the Min-MBR-All (R = 0.35, p < 0.0001) were age (β = −0.22, p < 0.0001), heart rate (β = 0.21, p < 0.0001) and the RBC count (β = −0.17, p < 0.0001). Because the correlation coefficients (r) of MABP and OPP, weight, BMI, and waist circumference were over 0.8, we excluded MABP and waist circumference from the explanatory variables.

**Table 3:** Correlation coefficients from the univariate regression analysis between Max MBR-All, Min MBR-Vessel, Min MBR-All, and all parameters.

Objective variable: Explanatory variables	Max MBR-All	Min MBR-Vessel	Min MBR-All
	r	r	r
Height	−0.24^⁂^	−0.01	−0.08^*^
Weight	−0.25^⁂^	0.06	−0.09^⁑^
Waist circumference	−0.17^⁂^	0.03	−0.07*
BMI	−0.16^⁂^	0.07*	−0.06
Heart rate	−0.06	0.18 ^⁂^	0.20^⁂^
MABP	−0.16^⁂^	−0.01	−0.08*
Pulse pressure	0.11^⁑^	−0.08*	−0.07*
Spherical refraction	0.12^⁑^	0.07*	0.02
IOP	0.03	0.01	0.02
OPP	−0.17^⁂^	−0.01	−0.09^⁑^
WBC	−0.07*	0.07*	0.004
RBC	−0.31^⁂^	−0.001	−0.14^⁂^
Platelets	−0.03	0.06	0.04
eGFR	0.10^⁑^	0.03	0.10^⁑^
Current smoking	0.01	0.09^⁑^	0.08*
(+ = 1, − = 0)			

*p < 0.05, ^⁑^p < 0.001, ^⁂^p < 0.0001.

**Table 4:** Results of multivariate regression analyses for factors independently contributing to Max MBR-All, Min MBR-Vessel, and Min MBR-All.

Objective variable: Explanatory variables	Max MBR-All	Min MBR-Vessel	Min MBR-All
	*β*	*β*	*β*
Male = 1, Female = 0	−0.15^⁑^	−0.09^⁑^	−0.05
Age		−0.25^⁂^	−0.22^⁂^
Height	0.003		−0.02
Weight	−0.05		0.01
BMI		0.09*	
Heart rate		0.17^⁂^	0.21^⁂^
MABP			
Pulse pressure	0.13^⁑^	−0.04	−0.01
Spherical refraction	0.08*	0.14^⁂^	
OPP	−0.14^⁑^		−0.01
WBC	0.01	0.01	
RBC	−0.17^⁂^		−0.17^⁂^
eGFR	0.05		
Current smoking		0.06	0.01
(+ = 1, − = 0)			

R = 0.40, 0.35, 0.35, p < 0.0001, respectively.

*p < 0.05, ^⁑^p < 0.001, ^⁂^p < 0.0001.

## Discussion

Our previous study revealed that the average MBR-Tissue and average MBR-All values obtained by a morning evaluation in patients with coronary artery disease were significantly lower than those of patients without coronary artery disease^11^. It has also been clarified that in patients with diabetes mellitus, intima-media thickening shown by ultrasonography reflects a decrease in the average values of all sections of the MBR in the ONH^12^. In a study of glaucoma patients, the average MBR-All in patients with normal tension glaucoma was significantly lower than that of normal subjects^13^. In addition, the visual field mean deviation in patients with open angle glaucoma was significantly positively correlated with the average values of all sections of the MBR^14^.

Considering the above-cited reports, it appears that a low average MBR value in the ONH may reflect an unfavorable systemic or ophthalmic status. However, the average MBR is the mean value of the variation of the MBR during the period from systolic to diastolic during a cardiac cycle; in other words, a decrease in the average MBR is caused by a decrease in the Max MBR and/or Min MBR during a cardiac cycle. We hypothesized that there are important differences among the decreases in the average MBR in the ONH that are due to gender differences, those due to aging, and those due to systemic or ophthalmic disorders. The purpose of the present study was thus to clarify the nature of the influences of gender differences and aging on the ONH microcirculation, by determining the Max, Min, and average MBR values of healthy subjects.

A survey of healthy Japanese subjects revealed that the MABP in the men was higher than that in the women until the age of 60 years, whereas among the subjects >50 years old the pulse pressure in the women was higher than that in the men^15^. In our present study, the mean age of the men was 50.0 years and that of the women was 49.8 years. Because these ages reflect middle age, we speculate that the pulse pressure values in women have been higher than those of men (Table 1).

An epidemiologic survey in Japan showed that the rate of myopia in men tended to be higher than in women^16^, and a higher eGFR has been confirmed in women compared to men^17^. Herein we conducted a univariate regression analysis to determine the correlation coefficients among gender, age, and all sections of the MBR. As in previous investigations^6–9^, we observed that the average MBR-All was significantly negatively correlated with gender and age. However, the Max MBR-All was most strongly correlated with gender among all sections of the MBR. In addition, the Min MBR-Vessel (r = −0.24) was most strongly correlated with age, followed by the Min MBR-All (r = −0.20) among all sections of the MBR. The univariate analysis clarified that gender and age each influenced different parts of the MBR.

Our multivariate analysis clarified that gender influences the Max MBR and aging influences the Min MBR. These correlations were stronger than that of the average MBR. It has been suggested that the RBC volume influences both the maximum and the minimum MBR-All. Other research groups have confirmed that the RBC volume is a main contributing factor for the average MBR in the ONH^7^. The measurement of the RBC count is therefore essential for the measurement of ONH microcirculation by LSFG, as well as the measurements of spherical refraction and the ocular axial length.

Compared to average MBR-Tissue, Min MBR-Tissue was more strongly correlated with gender. In addition, average MBR-Tissue did not show a significant correlation with age, whereas Max MBR-Tissue was significantly positively correlated with age, and Min MBR-Tissue was significantly negatively correlated with age. However, the multivariate regression analysis did not identify gender or age as an independent contributing factor for Min MBR-Tissue or Max MBR-Tissue. These findings suggest that compared to the other sections, gender and age have limited effects on MBR-Tissue, which supplies the blood flow to the ONH. The term MBR-Vessel represents large vessels that pass through the ONH to supply the retina. MBR-All is a blend of the MBR-Vessel and MBR-Tissue, and although MBR-Tissue has a much lower flow rate compared to MBR-Vessel, the latter still predominates. Clinicians and researchers using LSFG measurements should aware of its fact is an important matter.

We next addressed the question of which phenomena might be favorable for increasing the Max MBR or Min MBR in the ONH, based on our results. A low OPP value is a well-known risk factor for the development and progression of open angle glaucoma^18–21^. Pulse pressure represents vascular resistance, and it was reported that increasing pulse pressure is a significant risk factor for the extent of coronary artery disease^22^. In addition, our previous study also revealed that pulse pressure reflects peripheral vascular resistance, which is represented by the pulse waveform parameter in the ONH^23^. In other words, the variation of the MBR of the ONH in the cardiac cycle expands in parallel with an increase in pulse pressure.

Vascular resistance is recognized as a main pathogenesis for retinal vein occlusion, which is a common retinal vascular disease^24,25^. It was reported that cardiovascular diseases are more frequent in men^26^; however, no significant difference in the development of retinal vein occlusion between the genders has been reported^27,28^. We observed herein that aging led to decreases in the values of Min MBR-Vessel and Min MBR- All. The human arterial system in youth is designed for its role of receiving spurts of blood from the left ventricle and distributing this as a steady flow through peripheral capillaries^29^. In older individuals, pulsations are not absorbed in the large arteries, and they therefore extend down into the microcirculation^29^. We speculate that an upper part of the steady blood flow of the ocular microcirculation may be represented by the Min MBR. From the viewpoint of steady flow, we suspect that for the goal of improving systemic and ophthalmic conditions, increasing the Min MBR is more favorable than increasing the Max MBR.

**Figure 3:**
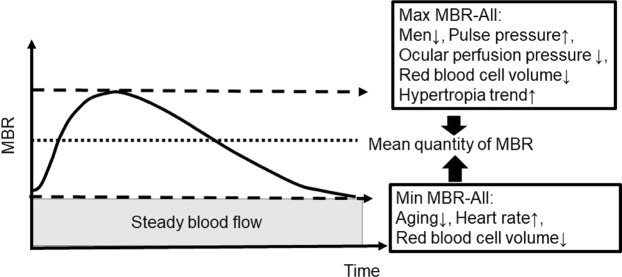
Hypothesis regarding the influences of aging, gender, ophthalmic status, and systemic hemodynamics on the entire ONH circulation in healthy adults.

Figure 3 illustrates our hypothesis regarding the roles of aging, gender, ophthalmic status, and systemic hemodynamics in the ONH microcirculation in healthy adults. Evaluations of only the average MBR may lead to a misunderstanding of the significance of the variation in ONH microcirculation. We propose that it is important to take into account the variations of both the Max MBR and the Min MBR, rather than only the average MBR. A high heart rate can maintain a steady ocular blood flow, but a heart rate that is too high is recognized as a risk factor for cardiovascular disease^30,31^. A further study is needed to reexamine the ocular hemodynamics in patients with high heart rates. In any case, it is necessary to clarify the significance of the increase or decrease of Min MBR by using physiological parameters such as the PWV and methods such as carotid ultrasonography and echocardiography. Our present findings provide important information about the details of the ONH microcirculation.

Our study has some severe limitations. First, because the participants of the medical checkup program were more often men than women, there was a clear imbalance in the numbers of male and female subjects (n = 701 vs. n = 208). Second, it was reported that lens opacity may underestimate hemodynamic quantifications obtained by LSFG^32^. We excluded subjects whose best corrected visual acuity was <40/50 and showed blur fundus by camera. However, the possibility that lens opacity was present in a few subjects cannot be completely ruled out. Third, the correlation coefficients revealed by the multivariate regression analysis were not very high. Further research is necessary to identify novel contributing factors for Max and Min MBR. Finally, we did not evaluate more commonly used tools for examining microcirculation, such as optical coherence tomography angiography. Careful validation studies with larger patient populations of both genders and including subjects from different age groups (e.g., adolescent, young adult, middle-aged, and elderly subjects) with various systemic conditions are needed.

To conclude, the results of our analyses demonstrated that gender and aging influenced different parts of the MBR in manner that are distinct from the influence of the average MBR. Our findings clarified that gender differences influence the Max MBR and aging influences the Min MBR, and these correlations were stronger than that of the average MBR.
